# Lissencephaly-1 dependent axonal retrograde transport of L1-type CAM Neuroglian in the adult drosophila central nervous system

**DOI:** 10.1371/journal.pone.0183605

**Published:** 2017-08-24

**Authors:** Sirisha R. Kudumala, Tyrone Penserga, Jana Börner, Olesya Slipchuk, Priyanka Kakad, LaTasha H. Lee, Aater Qureshi, Jan Pielage, Tanja A. Godenschwege

**Affiliations:** 1 Department of Biological Sciences, Florida Atlantic University, Jupiter, Florida, United States of America; 2 Harriet L. Wilkes Honors College, Florida Atlantic University, Jupiter, Florida, United States of America; 3 Department of Biology, Division of Zoology/Neurobiology, Technische Universität Kaiserslautern, Kaiserslautern, Germany; Universitat Regensburg, GERMANY

## Abstract

Here, we established the Drosophila Giant Fiber neurons (GF) as a novel model to study axonal trafficking of L1-type Cell Adhesion Molecules (CAM) Neuroglian (Nrg) in the adult CNS using live imaging. L1-type CAMs are well known for their importance in nervous system development and we previously demonstrated a role for Nrg in GF synapse formation. However, in the adult they have also been implicated in synaptic plasticity and regeneration. In addition, to its canonical role in organizing cytoskeletal elements at the plasma membrane, vertebrate L1CAM has also been shown to regulate transcription indirectly as well as directly via its import to the nucleus. Here, we intend to determine if the sole L1CAM homolog Nrg is retrogradley transported and thus has the potential to relay signals from the synapse to the soma. Live imaging of c-terminally tagged Nrg in the GF revealed that there are at least two populations of retrograde vesicles that differ in speed, and either move with consistent or varying velocity. To determine if endogenous Nrg is retrogradely transported, we inhibited two key regulators, Lissencephaly-1 (Lis1) and Dynactin, of the retrograde motor protein Dynein. Similar to previously described phenotypes for expression of poisonous subunits of Dynactin, we found that developmental knock down of Lis1 disrupted GF synaptic terminal growth and that Nrg vesicles accumulated inside the stunted terminals in both mutant backgrounds. Moreover, post mitotic Lis1 knock down in mature GFs by either RNAi or Clustered Regularly Interspaced Short Palindromic Repeats (CRISPR) induced mutations, resulted in normal length terminals with fully functional GF synapses which also exhibited severe accumulation of endogenous Nrg vesicles. Thus, our data suggests that accumulation of Nrg vesicles is due to failure of retrograde transport rather than a failure of terminal development. Together with the finding that post mitotic knock down of Lis1 also disrupted retrograde transport of tagged Nrg vesicles in GF axons, it demonstrates that endogenous Nrg protein is transported from the synapse to the soma in the adult central nervous system in a Lis1-dependent manner.

## Introduction

L1-type CAMs are plasma membrane proteins that are well known for their role in nervous system development. Mutations in human L1CAM are linked to psychiatric diseases like schizophrenia, autism and a broad spectrum of neurological disorders called CRASH syndrome [1–10]. We previously uncovered novel mechanisms for the sole invertebrate L1-type CAM Neuroglian (Nrg) in transsynaptic signaling. We showed that it is critical for synapse growth and stability and that its function in organizing the cytoskeleton at the synapse is conserved from flies to humans [11–13]. In the adult L1-type CAMs have also been implicated in synaptic plasticity, memory formation as well as in regeneration after traumas [14]. The L1CAM signaling pathways in these cellular processes involve both, local signaling involving cytoskeleton rearrangements as well as alteration of transcription via extracellular signal–regulated kinases (ERK) and Nuclear Factor-κB (NF-κB) signaling [14, 15]. In addition, three distinct fragments of proteolytically cleaved L1CAM have also been shown to translocate to the nucleus and regulate expression of genes with functions in migration, DNA post replication repair, cell cycle control and differentiation [16–19]. However, the relay mechanism of L1CAM signaling pathways from the synapse to the soma remain to be elucidated. Here, we intend to determine if Nrg is retrogradely transported from the synapse in the adult and thus has the potential to serve as a direct or indirect signaling molecule in the soma in addition to its canonical function at the plasma membrane.

Currently only few transmembrane proteins have been reported to be transported in “signaling endosomes” in a retrograde fashion, such as bone morphogenetic protein (BMP), neurotrophin and purinergic receptors [20–23]. While some cytoplasmic cargos bind directly to microtubule based motors, most cargo proteins appear to require adaptor proteins. For example APP binds JNK-interacting protein 1 (Jip1) which in turn regulates kinesin and dynein activity [24, 25]. Jip3 was found to regulate retrograde transport of activated c-Jun N-terminal Kinase (JNK) and lysosomes but not endosomes and autophagosomes [26]. Finally, Snapin was shown to recruit dynein to BDNF-TrkB signaling endosomes for retrograde transport [27].

Retrograde transport requires microtubule-associated motor Dynein, which moves towards the minus ends of microtubules. The multiprotein complex Dynactin, with the major 150K subunit being an orthologue of Drosophila *glued*, is a dynein activator that binds to phospholipid vesicles via spectrin/arp1 and AnkB to promote fast axonal transport of vesicles, mitochondria, endosomes, and lysosomes as well as has a function in initiation of retrograde transport [28–33]. Dynactin also interacts with anterograde motor kinesin-2 allowing bi-directional transport of organelles and vesicles [34]. Similar to Dynactin, Lissencephaly-1 (Lis1) also regulates Dynein [28, 35]. Together with NudEL the Lis1/NudEL/dynein complex was shown to be required for retrograde transport of high-load cargo, such as mitochondria and endosomal vesicles and has a function in cargo initiation as well [36–42]. While the coordination of dynactin and Lis1 in dynein regulation is not yet fully understood in axonal retrograde transport, they appear to have antagonistic roles in spindle assembly [43].

Expression of a poisonous subunit of dynactin p150 Glued protein (Glued^Δ96B^), has previously been shown to disrupt Giant fiber (GF) synapse formation in Drosophila [44]. Here, we characterize the synaptic phenotypes of the GFs in Lis-1 knock down animals as well as axonal trafficking of Nrg in the adult to determine if L1-type CAMs are retrogradley transported from the synaspe to the soma.

## Materials and methods

### Fly stocks

The *w*^*1118*^ (wild type control, #38323), *lis1*^*G10*.*14*^ (null mutant, #64140), *lis1*^*k11702*^ (hypomorph, #10179), *lis1*^*G10*.*14*^/CyO;P{UAS-Lis-1.L}3 (#64140), P{TRiP.HMS01457 (#35043, referred to as UAS-Lis1^RNAiH^), P{KK108813} (#v106777, referred to as UAS-Lis1^RNAiK^), UAS-CD8-GFP (#5137), P{GMR91H05-GAL4}attP2 (#40594, referred to as R91H05), P{GMR68A06-GAL4} (#39449, referred to as R68A06), P{UAS-Cas9.P}attP2 (#54595, referred to as UAS-Cas9), and P{GawB}OK307 (#6488, referred to as A307) stocks were obtained from the Bloomington Stock Center (Indiana, USA) and the Vienna Drosophila Resource Center (Vienna, Austria). UAS-Nrg^eGFP^ (inserted at attP40), UAS-Nrg^mCherry^ (inserted at attP40), GF-Split-Gal4, UAS-Glued^Δ96^, UAS-Lis1^gRNA^ and P{UAS-Lis1.3xEmeraldGFP} (referred to as UAS-Lis1^GFP^) have been previously described and were obtained from the Card, Murphey, Bullock and Pielage labs [11–13, 44–49]. R91H05 and A307 were used to drive construct expression in the GF throughout its development, while A307 but not R91H05 drives expression also in the postsynaptic targets of the GF [50–52]. R68A06 and GF-Split-Gal4 drive expression in the GF but not its postsynaptic targets [47, 51] and we determined that they turn on expression after the GF synaptic terminals have formed at around 60% and 90% of pupal development, respectively [53, 54]. All genetic crosses were performed on standard fly media at 25°C and 2–5 days old flies were used for all of the experiments unless indicated otherwise.

### GF preparation and immunohistochemistry

The procedure for adult Drosophila nervous system dissection and subsequent dye-injection has been previously described in detail [55, 56]. To visualize the morphology of the GF-TTMn connection either a 10 mM Alexa Fluor 555 Hydrazide (Molecular Probes) in 200 mM KCl or a dye solution of 10% w/v neurobiotin (Vector Labs) and tetramethylrhodamine isothiocyanate (TRITC)-dextran (Invitrogen) in 2 M potassium acetate was injected into the GF axons by passing hyperpolarizing or depolarizing current, respectively. Preparation of GF samples for confocal microscopy has been described previously [55, 56]. The following antibodies and concentrations were used: Streptavidine-Cy3 conjugate (Jackson; 1:750 dilution), BP104 (Developmental Studies Hybridoma Bank, 1:50 dilution), goat anti-mouse-Cy2 and Cy5 (Jackson ImmunoResearch, 1:500 dilution), anti-GFP A11122 (Invitrogen, 1:500 dilution). Samples were scanned at a resolution of 1024x1024 pixels, 2.5x zoom, and 0.5 μm step size with a Nikon C1si Fast Spectral Confocal system using a 60×/1.4 NA oil immersion objective lens. Images were processed using Nikon Elements Advance Research 4.4 and Adobe Photosuite CS5 software.

### Electrophysiological recordings from the GF circuit

The procedure of electrophysiological recordings from the GF circuit has been described in detail previously [57]. The flies were given ten single pulses at 30–60 V for 0.03 ms with a 5 second interval between the stimuli and the shortest response latency of each fly was averaged. To determine the reliability of the circuit, ten trains of ten stimuli were given at 100 Hz with an interval of 2 seconds between the trains and the percent of the total responses was calculated. All of the traces were recorded, stored and analyzed using pClamp 10 (Molecular Devices) software. Mann-Whitney Rank sum tests were used to determine significant differences (* p ≤ 0.05, ** p ≤ 0.01, *** p ≤ 0.001) in average response latencies and following frequencies between different genotypes (Sigma Plot 11 software).

### Live imaging and analysis

The CNS of 1–3 day old animals expressing tagged Nrg reared at 25°C were dissected in saline (NaCl 128 mM, KCl 2 mM, CaCl_2_ 1.8 mM, MgCl_2_ 4 mM, HEPES 5mM, sucrose 35.5 mM, pH = 7.2) and mounted on poly-lysine coated glass slides. The GF axons were imaged at the dorsal side in the cervical connective (CvC) with a Nikon A1 or a Nikon A1 plus Confocal with GaAsP multi-detector units using a CFl Plan 100x/1.1 NA water immersion lens or a CFI Plan APO lamba 60x/NA1.4 oil objective at room temperature (20–23°C). The 488nm or the 561nm excitation line was used at 50–100% of power for photobleaching (20–30 sec) and between 2–10% of its maximum power for live imaging. Images were acquired at 1 to 4 frames per second for up to 10 minutes. Nikon Elements Advanced Research 4.4 was used for video analyses and kymograph generation. Anterograde and retrograde vesicles entering the bleached area were scored and the flux was expressed as the total vesicle number per minute. In addition, we determined the velocity distribution for anterograde and retrograde vesicles using Nikon Elements Advanced Research 4.4 and Microsoft Excel 2013. For velocity we scored vesicle runs that included velocity changes. For this we determined the maximum net displacement in either antero- or retrograde direction during the imaging period of a vesicle and calculated the average travel speed in μm/second. Stationary vesicles were counted in unbleached regions of the axon and the average was calculated per μm axon length. Kruskal-Wallis One Way Analysis Of Variance and Mann-Whitney Rank Sum Test (Sigma Plot 11 software) were used to determine significant differences (* p ≤ 0.05, ** p ≤ 0.01, *** p ≤ 0.001).

## Results

### Trafficking of tagged Nrg vesicles in GF axons

**Fig 1:**
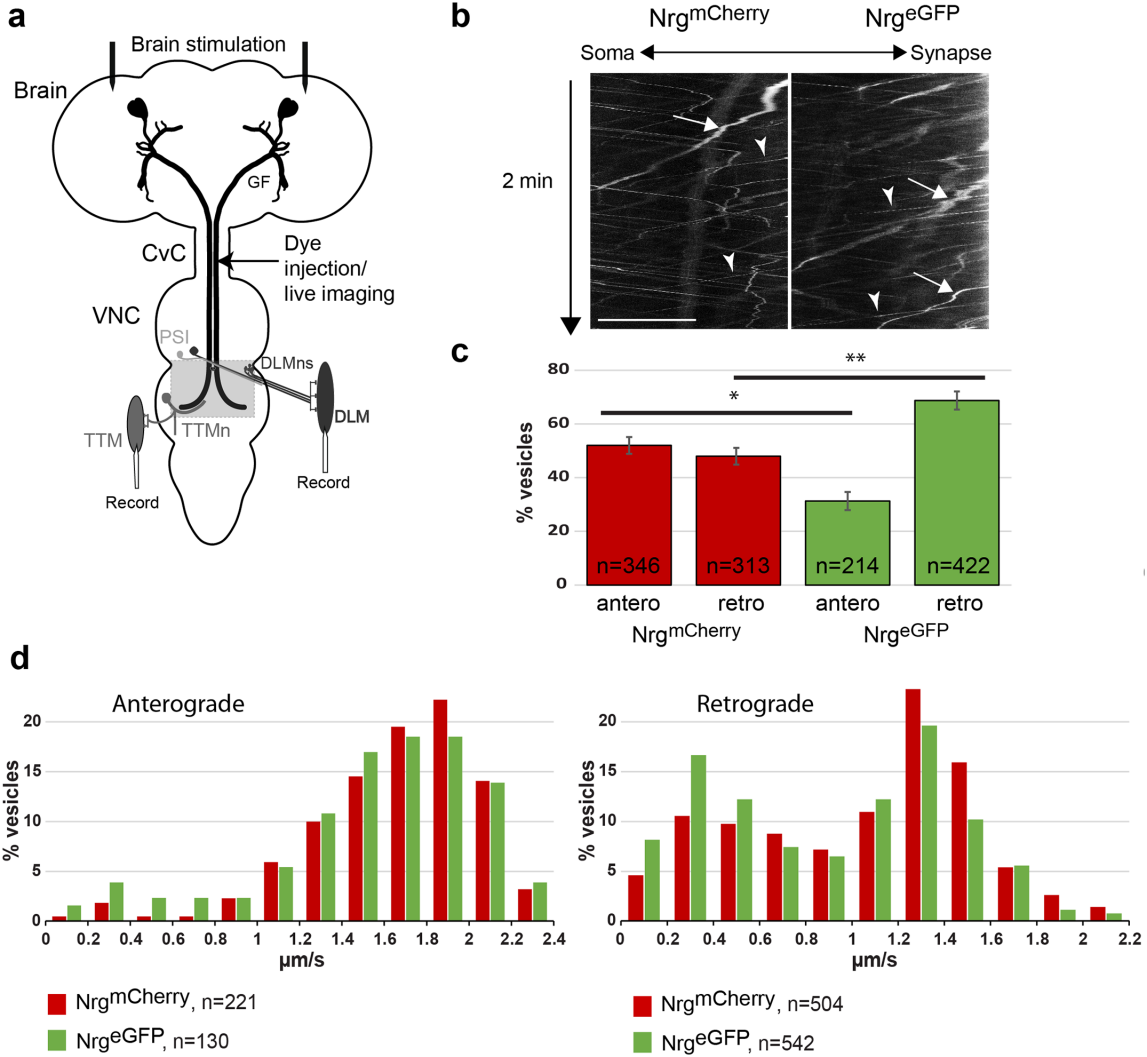
Trafficking of tagged-Nrg vesicles in the GF axon. (a) Schematic representation of the GFC in the nervous system. Both GFs as well as the downstream circuit with the TTMns, the PSIs and the dorsal longitudinal motor neurons (DLMns) for one GF are depicted. DLMns and TTMns synapse onto the dorsal longitudinal muscles (DLMs) and TTMs, respectively. Live imaging and dye injections of GF axon for visualization of the GF anatomy were performed in the cervical connective (CvC). Areas of anatomical analysis by confocal microscopy in the ventral nerve cord (VNC) are highlighted by a gray square. Placement of stimulation and recording electrodes for electrophysiological analysis are shown. (b) Kymographs of mCherry- and eGFP-tagged Nrg vesicles in GF axons of homozygous UAS-Nrg^eGFP^;R68A06 and UAS-Nrg^mCherry^;R68A06 flies. Axons were imaged in the CvC at 4 frames/second. A small region of the axon was photobleached before acquisition to better visualize vesicle trafficking. Ascending and descending slopes represent retrograde and anterograde vesicles, respectively. Arrows point to bright, slow moving, while arrowheads indicate fast moving retrograde vesicles. Scale bar is 10 μm. (c) Directionality of anterograde and retrograde Nrg^eGFP^ and Nrg^mCherry^ vesicles. Numbers of vesicles (n) statistically (Mann-Whitney Rank Sum Test, * p = 0.013 and **p = 0.007) analyzed in ten axons were expressed in percent of total vesicles. (d) Frequency distribution of velocities of anterograde and retrograde mCherry- and eGFP-tagged Nrg vesicles. The numbers of vesicles (n) for velocity increments of 0.2 μm/sec in five axons was calculated in percent.

Here we established the GF neurons as a novel model to study axonal trafficking in the adult CNS. The two GFs, each with an axon diameter of 5–8 μm, are the command neurons of the giant fiber circuit (GFC), which mediate the escape response of the fly (Fig 1a) [58]. The GF cell body and dendrites are located in the brain and the axon synapses with the tergotrochanteral motoneuron (TTMn) and the peripherally synapsing interneuron (PSI) in the ventral nerve cord (VNC, Fig 1a). The TTMn innervates the tergotrochanteral muscle (TTM, Fig 1a).

We used R68A06 Gal4 line, which selectively expresses in the GFs but not in the pre- or postsynaptic neurons of the GF circuit, to visualize c-terminally eGFP or mCherry-tagged Nrg axonal trafficking. Both tagged transgenes are inserted at the same chromosomal location [11, 49], which ensures similar expression strength, and to maximize expression we used established stocks that are homozygous for the Gal4 driver and the UAS-constructs. We live imaged the GF axons in the CvC of one to four day old adult nervous systems. Like endogenous Nrg, tagged Nrg^eGFP/mCherry^ is strongly expressed at the axonal plasma membrane of mature GFs and to better visualize vesicle trafficking we photobleached a small section of the axons. Tagged Nrg vesicles moved in anterograde as well as in retrograde direction in both, bleached and unbleached, regions of the GF axon (Fig 1b) and we did not observe any difference in vesicle behavior between the regions (S1 and S2 Movies). With Nrg^mCherry^ we observed a similar amount of anterograde and retrograde vesicles moving into the bleached area per minute (Fig 1b and 1c), with on average 52% ± 3 vesicles moving into the anterograde direction. However, with Nrg^eGFP^ more retrograde and less anterograde vesicles appeared to be moving into the bleached area when compared to Nrg^mCherry^ (Fig 1b and 1c) with only 31% ± 3 of the vesicles being visible in anterograde direction. However, it should be noted that most of the anterograde Nrg^eGFP^ are exceedingly faint and barely visible in kymographs. This suggests that many of anterograde moving Nrg^eGFP^ vesicles are below optical resolution, which in turn would impacts the relative proportion of retrograde vesicles when calculated as the total number of vesicles in both direction.

To further compare eGFP and mCherry tagged vesicles during axonal transport, we determined the frequency distribution of velocities of anterograde and retrograde mCherry- and eGFP-tagged Nrg vesicles (Fig 1d). With the exception of few, almost all anterograde Nrg^eGFP^ and Nrg^mCherry^ vesicles moved at constant speed without any major stops in the imaged area of about 20–25 μm (Fig 1b). The majority of vesicles had a velocity between 1.6 μm/s and 2 μm/s and there was no statistically significant difference in the average speed of all vesicles (Nrg^mCherry^ 1.67 μm/s ± 0.03 and Nrg^eGFP^ 1.55 μm/s ± 0.02, p = 0.058). In contrast to anterograde vesicles, there appeared to be at least two major populations of retrograde Nrg^eGFP^ and Nrg^mCherry^ vesicles. There are bright, slow moving retrograde vesicles that frequently changed their velocity during runs (Fig 1b, arrows) and the majority of these vesicles had an average velocity between 0.2 and 0.4 μm/s in the imaged area (Fig 1d). In addition, there are fast moving retrograde vesicles that moved with a more consistent velocity (Fig 1b, arrowheads) between 1.2 μm/s and 1.4 μm/s (Fig 1d). The average velocity of all retrograde vesicles was significantly different (Nrg^mCherry^ 1.05 μm/s ± 0.03 and Nrg^eGFP^ 0.91 μm/s ± 0.09, p<0.001), due to that less fast and more slow moving vesicles were seen with Nrg^eGFP^ when compared to Nrg^mCherry^ (Fig 1d). This suggests that the tags do not affect the actual speed of individual moving vesicles distinctively but the quantity of visible slow and fast moving vesicles.

In summary, the finding that both, Nrg^eGFP^ and Nrg^mCherry^ vesicles, are retrogradely transported, demonstrates that Nrg protein can be retrogradely transported. However, the differences between Nrg^eGFP^ and Nrg^mCherry^ vesicles suggests that either tag may not accurately reflect axonal trafficking of endogenous Nrg protein. Therefore, we analyzed Nrg in mutants that are known to disrupt retrograde transport, in which Nrg would be expected to accumulate in synaptic terminals if it is indeed retrogradely transported.

### Lis-1 knock down disrupts GF terminal development and Nrg localization

Lis1 and Dynactin are two distinct regulators of dynein that when mutated disrupt retrograde transport. Inhibition of Dynactin function by the expression of UAS-Glued^Δ96B^ has been previously shown to disrupt GF synapse formation, resulting in stunted terminals that often exhibited large vacuoles [44]. Here, we characterized Lis1 mutants to determine if they result in similar phenotypes as seen with the expression of Glued^Δ96B^ and if Nrg localization is affected in both mutant backgrounds.

**Fig 2:**
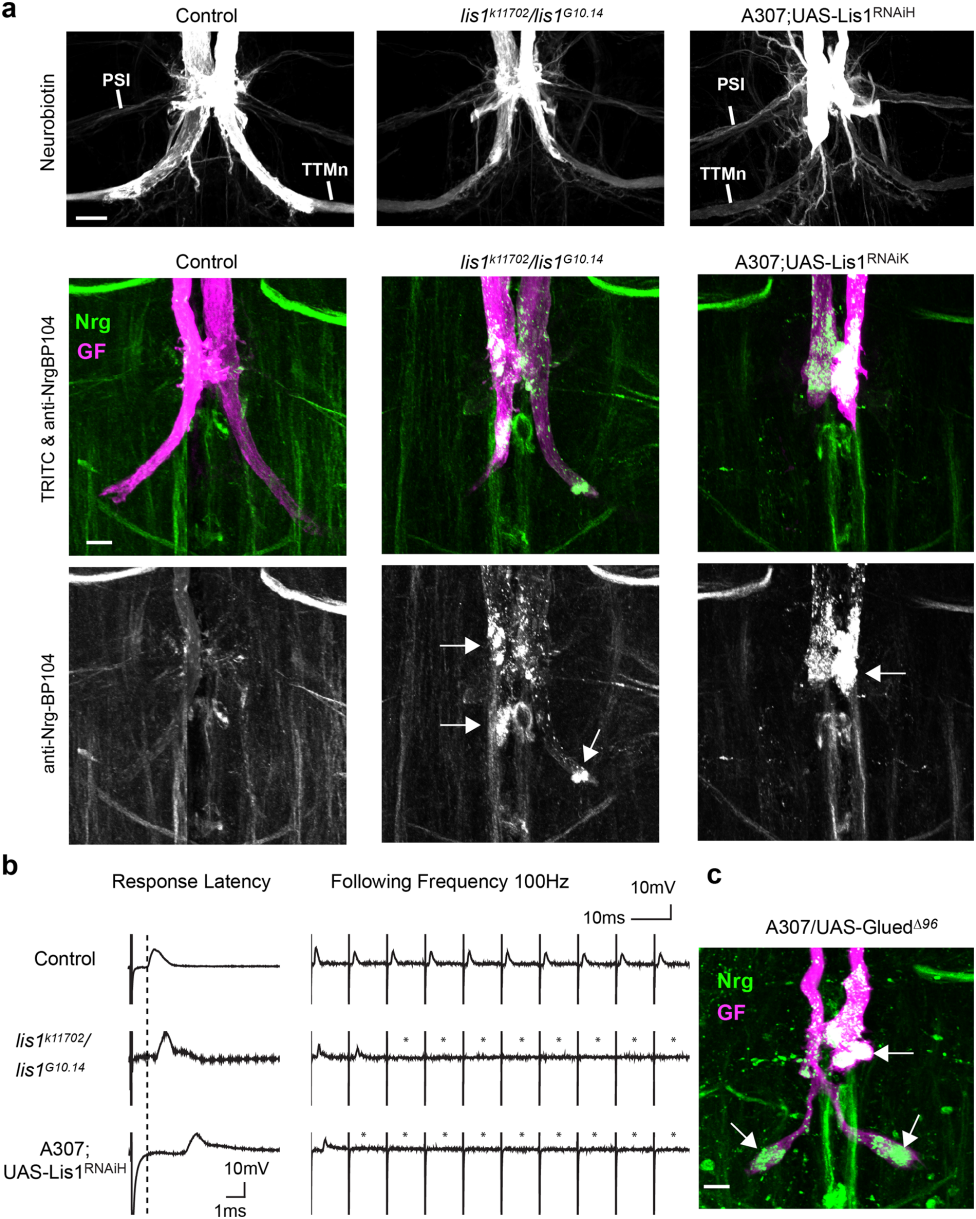
Lis1 phenotypes and Nrg localization in wild type and *lis1* mutant backgrounds. (a) Maximum intensity projection views of confocal image stacks showing representative GF terminals. GF synapse anatomy was visualized with neurobiotin (top panel) and TRITC-dextran (middle panel, magenta). Neuronal Nrg^180^ (green) was immunohistochemically labeled with monoclonal antibody BP104 (middle and lower panel). Neurobiotin dye-coupling of the GFs to the post-synaptic TTMns demonstrates the presence of synaptic connections between the medial TTMn dendrites and GF-TTMn. Accumulation of vesicular Nrg^180^ clusters in the synaptic terminal and in the axon at the PSI contact region are indicated with arrows. Scale bar is 10 μm. (b) Sample trace of electrophysiological recordings of the GF to TTM pathway from control animals and *lis1* mutants. The response latency of wild type animals is approximately 0.8 ms (dashed line). Ten stimuli given at 100 Hz and the lack of some responses in mutant animals are indicated with asterisks. (c) Maximum intensity projection view of confocal image stacks of GF terminal expression a poisonous subunit of Drosophila dynactin p150 Glued protein. GF was dye injected with TRITC-dextran (magenta) and Nrg^180^ (green) was immunohistochemically labeled with monoclonal antibody BP104.

The Drosophila *lis1* gene is located on the second chromosome and produces four annotated transcripts. The *lis1*^*G10*.*14*^ allele has a single point mutation (R239X) that leads to premature translation termination with no detectable Lis1 protein and results in lethality [46, 59–61]. However, trans-heterozygotes *lis1*^*k11702*^*/lis1*^*G10*.*14*^ (hereafter also referred to as hypomorphic *lis1* mutants) with the *lis1*^*k11702*^ allele, which has a P-element insertion in the 5’ untranslated region of the first exon of two of the four *lis1* transcripts, are viable [46, 59–61]. In addition, we used two RNAi lines, UAS-Lis1^RNAiH^ and UAS-Lis1^RNAiK^ with amplicons against the 6^th^ exon and in the 3’ untranslated region, respectively, to down regulate Lis1. For the anatomical characterization, we injected TRITC-dextran and neurobiotin dye mixtures into the GF axons to reveal the morphology of the terminals and to determine if the GFs are coupled to their synaptic targets via the gap junctions of their electrical-chemical synapses (Fig 2). In addition, we assessed the function of GF synapse using electrophysiology.

In *lis1*^*k11702*^/*lis1*^*G10*.*14*^ hypomorphs the GF terminals were shorter and thinner when compared to wild type controls, but still dye-coupled with the TTMn demonstrating the presence of a residual synaptic connection (Fig 2a). Correlating with the anatomical defects, the function of the GF to TTMn connection was disrupted in these hypomorphic mutants (Table 1). The response latencies were increased and the ability to follow 10 stimuli at 100 Hz was severely reduced (Fig 2b and Table 1). Ubiquitous expression of the Lis1 RNAi constructs was lethal, thus we used the A307 Gal4-line to drive expression in the GF and its postsynaptic target neurons throughout their development to determine if it would result in *lis1*^*k11702*^/*lis1*^*G10*.*14*^ like phenotypes. Both RNAi-lines resulted in indistinguishable anatomical phenotypes that were similar to the hypomorphic *lis1* mutants in that they disrupted GF synapse formation and its function but the morphological and functional phenotypes were even more severe (Fig 2 and Table 1).

**Table 1:** Average response latency and average following frequency of *lis1* mutants and control flies.

*Genotype*	*n*	*Average Response Latency in ms (ms ± SEM)*	*Average Percent Following Frequency in % (± SEM)*
A307/+	16	0.79±0.03	99±1
*lis1*^*k11702*^*/+*	14	0.95±0.06	95±4
*lis1*^*G10*.*14*^*/+*	20	0.82±0.02	99±1
*lis1*^*k11702*^*/lis1*^*G10*.*14*^	62	1.45±0.04	15±2
*lis-1*^*k11702*^/*lis1*^*G10*.*14*^; UAS-Lis1/R91H05	18	0.88±0.04	45±5
A307, *lis1*^*k11702*^*/lis1*^*G10*.*14*^;UAS-Lis1/+	20	0.86±0.03	97±3
UAS-Lis1^RNAiK^/+	28	0.88±0.02	97±1
UAS-Lis1^RNAiH^/+	24	0.97±0.04	96±2
R91H05/UAS-Lis1^RNAiH^	29	1.21±0.04	62±6
A307/UAS-Lis^RNAiK^	26	1.83±0.08*	11±1
A307/+;UAS-Lis1^RNAiH^ /+	24	2.56±0.18*	3±1
A307,UAS-CD8-GFP/+;UAS-Lis1^RNAiH^/+	22	2.14±0.12*	7±3
A307,UAS-Lis1^GFP^/+;UAS-Lis1^RNAiH^/+	16	0.87±0.06	100±0
A307,UAS-Lis1^GFP^/+	17	0.89±0.03	95±3
*w*^*1118*^, 1–5 days	10	0.82±0.02	99±1
*w*^*1118*^, 4–5 weeks	10	0.81±0.02	99±1
GF-Split-Gal4/+	10	0.80±0.02	99±1
GF-Split-Gal4/UAS-Lis1^RNAiH^, 1–5 days	24	0.78±0.02	96±2
GF-Split-Gal4/UAS-Lis1^RNAiH^, 4–5 weeks	16	1.05±0.09	74±10

*some animals exhibited no response when the GFs were stimulated in the brain und thus are not reflected in the average response latency calculation.

To determine Nrg localization in Lis1 and Glued^Δ96B^ mutant background, we used monoclonal antibody BP104 against the intracellular domain of Nrg. In wild type controls, Nrg labeling (Fig 2a, green) can only be seen faintly at the synaptic terminals of the GFs that were labeled with TRITC-dextran dye-injections (Fig 2a, magenta). In contrast, in *lis1* hypomorphic mutants and in animals expressing UAS-Lis1^RNAiH or K^ or UAS- Glued^Δ96B^ with A307, Nrg accumulated in vesicular clusters inside stunted synaptic terminals, as well as in the axon at the synaptic contact areas of the PSI (Fig 2a and 2c, arrows). In summary, our results suggest that Lis-1 has a function in GF synapse development and that both Lis1 and dynactin play a role in Nrg retrograde trafficking.

### Rescue of Lis1 phenotypes and Nrg localization

To demonstrate that the observed phenotypes are Lis1-specific and to determine spatial requirements for Lis1 in the GF circuit, we performed rescue experiments for both, *lis-1* hypomorphic mutants and Lis-1 RNAi knockdown animals.

**Fig 3:**
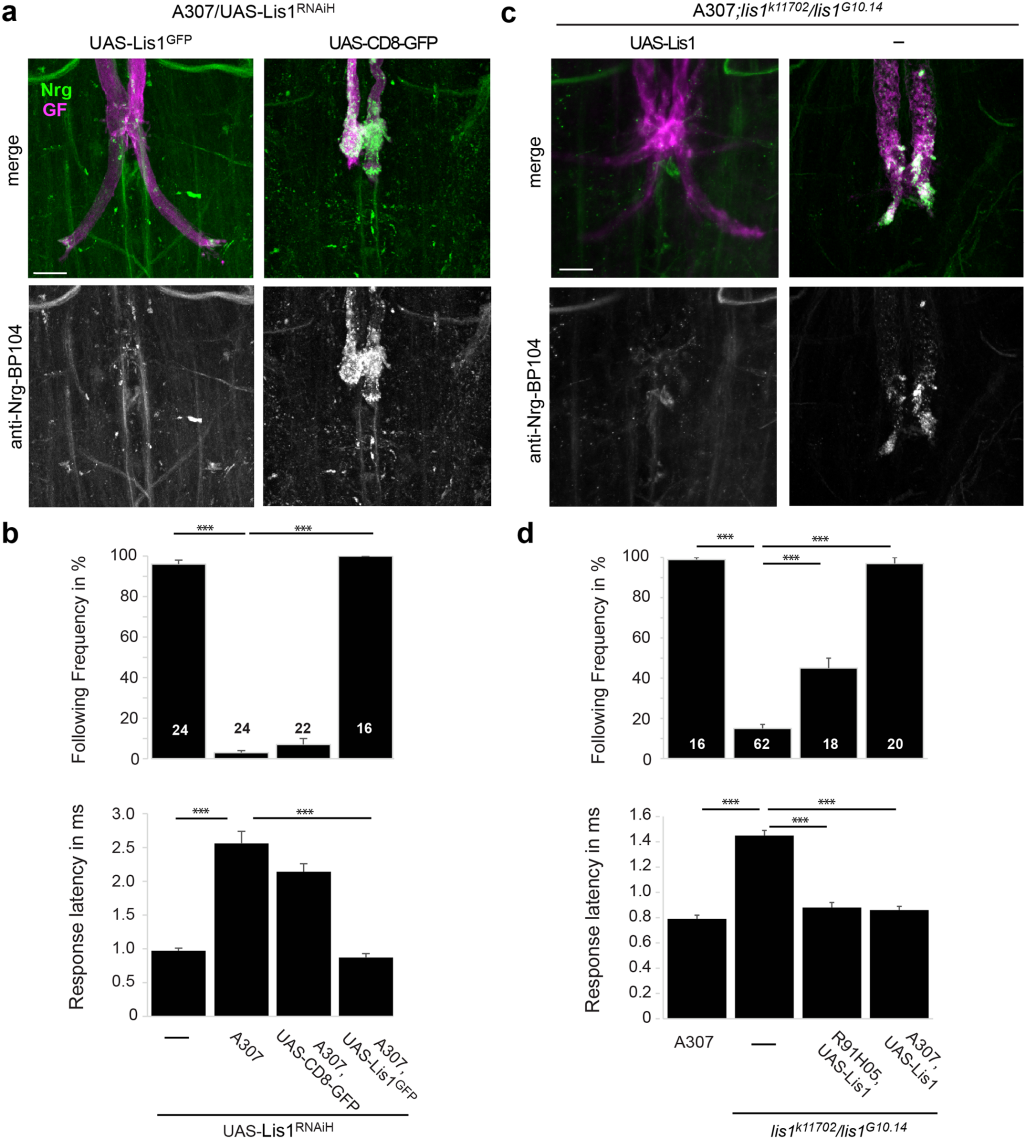
Rescue of Lis1 phenotypes. (a, b) Rescue of Lis RNAi phenotypes with co-expression of Lis1^GFP^. Co-expression of UAS-CD8-GFP with UAS-Lis1^RNAiH^ was used as a negative control. (c, d) Rescue of phenotypes in hypomorphic *lis1*^*k11702*^/*lis1*^*G10*.*14*^ with transgenic expression of wild type Lis1 in the GF and its target neurons or in the GF but not its target neurons with the A307 and R91H05 Gal4-drivers, respectively. A307, *lis1*^*G10*.*14*^*/lis1*^*k11702*^ without the UAS-Lis1 served as a negative control. (a, c) Giant fiber morphology and Nrg localization. All images show maximum intensity projections of confocal image stacks. GFs of adult flies were labeled by TRITC-Dextran injections (magenta) and displayed together with immunohistochemically labeled Nrg^180^ (anti-BP104 with goat anti-mouse-Cy5, pseudo-colored in green) in the VNC (upper row). Co-localization of both labels appears white. The lower row displays immunohistochemically labeled Nrg separately as gray scale images. Lis-GFP and CD8-GFP in (a) were present but are not shown. Scale bars are 20 μm. (b, d) The function of the GF synapse was determined with average following frequency and average response latency. Error bars indicate standard error of the mean and sample sizes are shown in the bars. Significant differences are indicated with asterisks (*** = p<0.001, Mann-Whitney Rank Sum Test).

**Fig 4:**
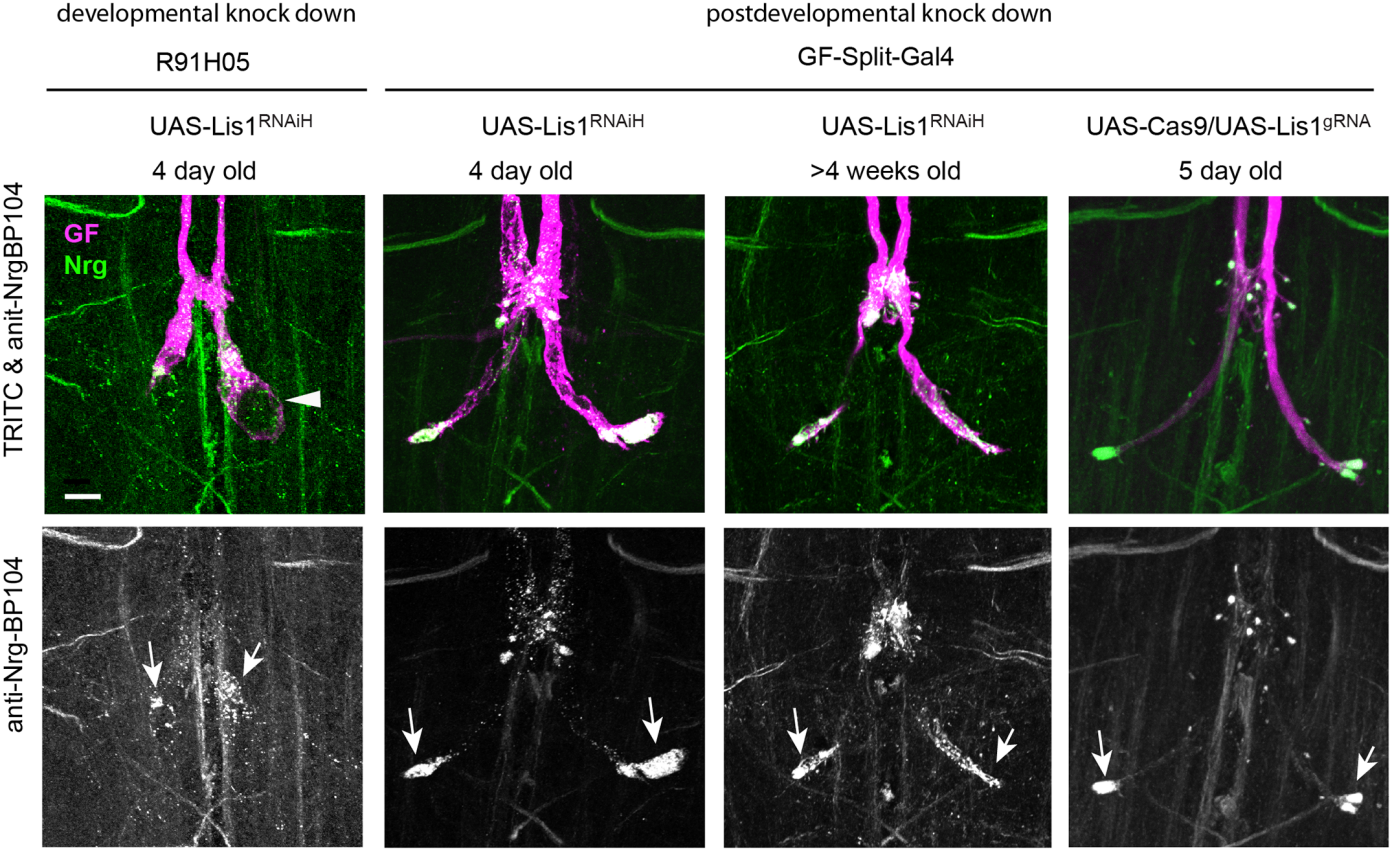
Phenotypes of GFs with post developmentally knocked down Lis1. Comparison of phenotypes when Lis1 was knocked down in the GF during its development (R91H05) or after its development (GF-Split-Gal4) with RNAi (UAS-Lis1^RNAiH^) and CRISPR (UAS-Lis1^gRNA^). All images show maximum intensity projections of confocal image stacks. GFs of adult flies were labeled by TRITC-dextran injections (magenta) and displayed together with immunohistochemically labeled Nrg^180^ (green) in the VNC (upper rows). Co-localization of both labels appears white. The lower rows display immunohistochemically labeled Nrg^180^ separately as gray scale images. Scale bars are 20 μm. Localization of Nrg^180^ (anti-Nrg-BP104, green) in wild type and *lis1* mutant backgrounds. Vesicular accumulations of Nrg^180^ in stunted and normal sized terminals are indicated by arrows. A large vacuole is indicated by an arrowhead.

The amplicon of the UAS-Lis1^RNAiH^ is directed against the 3’ untranslated region of the Lis1 gene, which is not present in the UAS-Lis1^GFP^ transgene [46]. Co-expression of UAS-Lis1^RNAiH^ with UAS- Lis1^GFP^ fully restored the GF anatomically as well as functionally and Nrg accumulation in the axon or terminal did not occur (Fig 3a and 3b, Table 1). To test if the rescue with UAS- Lis1^GFP^ expression was not the consequence of reduced expression of the RNAi transgene, we co-expressed membrane-bound GFP (UAS-CD8-GFP) with UAS-Lis1^RNAiH^ and we did not observe a rescue of any phenotypes (Fig 3a and 3b, Table 1). Similarly, we find that pre- and postsynaptic expression of wildtype Lis1 with the A307 driver was able to rescue all GF phenotypes observed in *lis1*^*k11702*^*/lis1*^*G10*.*14*^ mutants (Fig 3c and 3d, Table 1). Interestingly, with the R91H05 driver, which drives expression throughout GF development but not in its postsynaptic neurons, we were able to rescue the GF morphology and Nrg localization of *lis1*^*k11702*^*/lis1*^*G10*.*14*^ animals (data not shown) but only partially rescued the function of the GF to TTM pathway (Fig 3d and Table 1). This suggests that Lis1 may also contribute at the postsynaptic side in establishing a fully functional GF synapse. However, expression of Lis RNAi constructs with R91H05 was sufficient to effectively disrupt GF synapse formation and resulted in stunted, functionally defective terminals that exhibited Nrg accumulations, sometimes with large vacuoles as it has been described for Glued^Δ96B^ as well (Fig 4a and Table 1) [44]. In summary, our results demonstrate that the anatomical and functional GF defects as well as the accumulation of Nrg vesicles in the GF terminals seen in trans-heterozygous *lis1* mutants and in animals that express Lis1 RNAi constructs are due to reduced Lis1 function.

### Post developmental knock-down of Lis1 using RNAi and CRISPR

In GFs with developmental Lis knockdown, Nrg is likely to have accumulated in the stunted terminals as the result of inhibited retrograde transport. However, it cannot be excluded that Nrg may have accumulated as a secondary consequence of the failure to grow a full-sized synaptic terminal and thus, does not allow to determine if Nrg is also retrogradely transported from mature synaptic terminals. Therefore, we used two approaches, RNAi and CRISPR, to post developmentally knock down Lis1 expression and determine if Nrg accumulates in normally developed GF terminals. For this we utilized the GF-Split-Gal4 line, which drives expression exclusively in the GF [47] and turns on expression after the GF terminals are fully matured.

In contrast to developmental expression of UAS-Lis1^RNAiH^ with R91H05, expression with the GF-Split-Gal4 line resulted in morphologically and functionally normal GFs (Fig 4, Table 1) when 1–5 day old animals were assessed but large vesicular accumulations of Nrg were observed inside the large synaptic terminals as well as in the PSI contact regions (Fig 4, arrows). These results demonstrate that Nrg accumulations is not due to any disruption of the GF terminal development or function and that Nrg is retrogradely transported from the synapse to soma in the adult. Similarly, Nrg accumulation was still observed in 4 week old animals but the axon and synaptic terminal, although still present, appeared to be thinner (Fig 4a). This correlated with a significant increase of response latency when compared to 4 day old animals (p< 0.0001, Mann-Whitney Rank sum test) or 4 week old control wild type animals (p< 0.0056, Mann-Whitney Rank sum test, Table 1), which was associated with subtle but non-significant decrease in the ability of the GF-TTMn synapse to follow stimuli at 100Hz. These results suggest that Nrg retrograde transport form the Gf synapse is maintained throughout adulthood.

To determine if CRISPR can be employed to mutate the *lis1* gene in the mature GFs, we co-expressed UAS-Cas9 and UAS-Lis1^gRNA^ [48] with GF-Split-Gal4 as well as with R68A06 and determined Nrg localization in 5 day old adults. Similar to Lis1-RNAi expression, we found that with GF-Split-Gal4 (n = 6) and with R68A06 (n = 8) all GF terminals were normal in length but had Nrg accumulations at the terminal tips and the PSI contact regions (Fig 4). This demonstrates that CRISPR can be used to effectively mutate genes in the GF post mitotically and further confirms that endogenous Nrg is transported in a retrograde manner.

**Fig 5:**
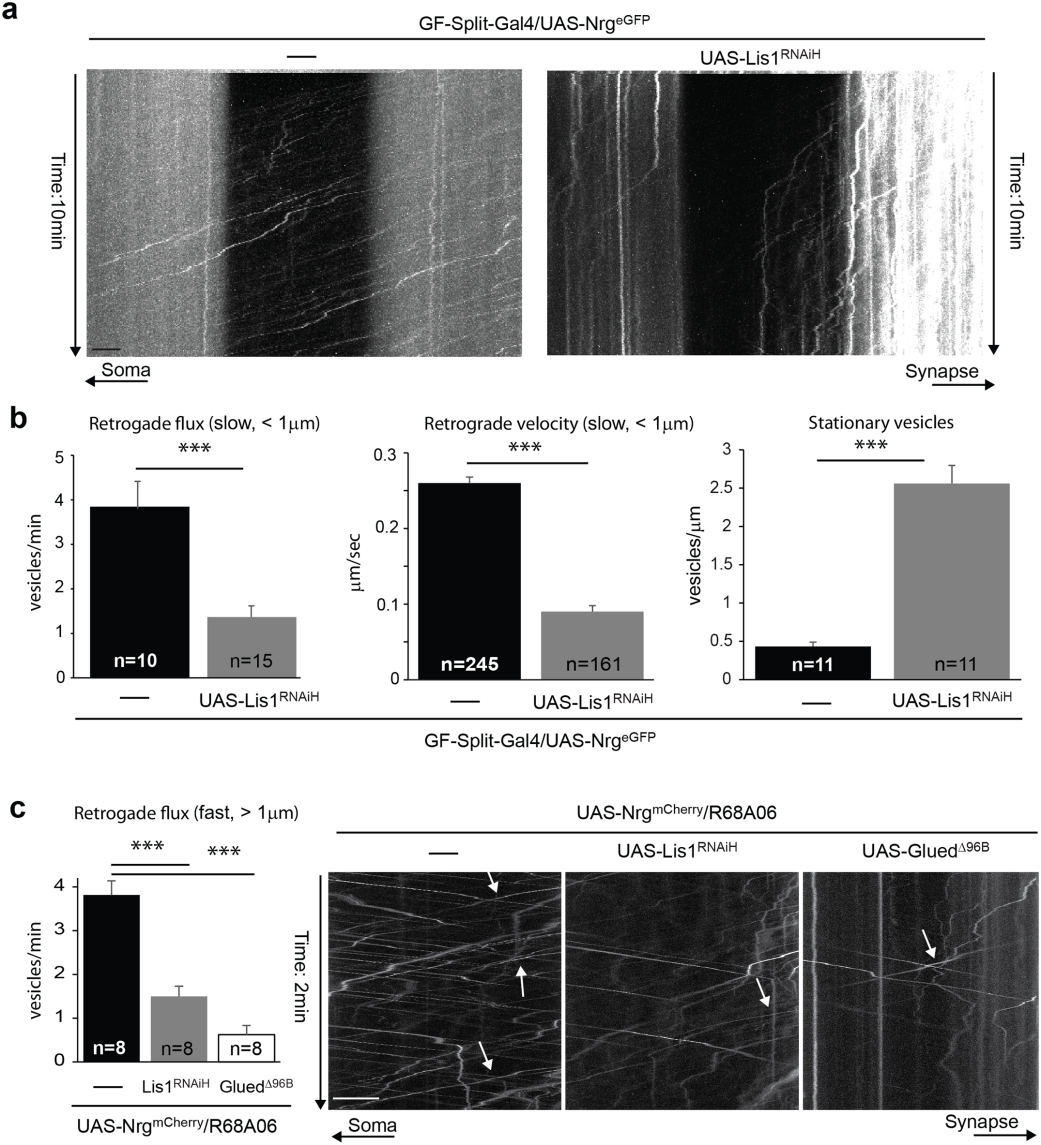
Retrograde trafficking of tagged Nrg in Lis1 knock down and Glued^Δ96B^ mutant background. (a) Axonal transport of Nrg^eGFP^ vesicles in GFs with postdevelopmental Lis1 knock down. Kymographs showing trajectory of Nrg^eGFP^ vesicles in a photobleached area flanked by unbleached areas in the GF axon over a 10 min time period. Videos were obtained at one frame per second. Ascending and descending slopes represent retrograde and anterograde vesicles, respectively. Vertical lines are stationary vesicles. Scale bar is 4 μm. (b) Quantification of Nrg^eGFP^ vesicles in wild type and in flies expressing UAS-Lis1^RNAiH^ with GF-Split-Gal4. The lower expression, magnification and one frame per second imaging rate allows to quantify only slow (< 1μm/sec) moving Nrg^eGFP^ vesicles. The average flux (left graph) are the average numbers of retrograde vesicles that entered the photobleached area per minute, with n indicating the number of GFs analyzed. The maximum net displacement of retrograde Nrg^eGFP^ vesicles during the imaging period was analyzed and the average travel velocity was expressed in μm/second. Ten and 15 axons were assessed in wild type and Lis1 knock down background, respectively, with n indicating the number of vesicles analyzed. The average number of stationary vesicles per micron axon length was assessed in the unbleached regions. A total axon length of 169 μm and 180 μm was assessed in wild type and Lis1 knock down background, respectively, with n indicating the number axons analyzed. Error bars show standard error of the mean and significant differences are indicated with asterisks (*** = p<0.001, Kruskal-Wallis One Way Analysis Of Variance and Mann-Whitney Rank Sum Test). (c) Expression of Nrg^mCherry^ without and with UAS-Lis1^RNAiH^ and UAS-Glued^Δ96B^ using the R68A06 driver line. Left panel, quantification of fast (> 1μm/sec) retrograde vesicles in eight (n) GF axons of each genotype. Error bars show standard error of the mean and significant differences are indicated with asterisks (*** = p<0.001, Kruskal-Wallis One Way Analysis Of Variance and Mann-Whitney Rank Sum Test). Right panel, kymographs of Nrg^mCherry^ vesicles without and with Lis1^RNAiH^ and Glued^Δ96B^ expression. Videos were obtained at 4 frames per second and a 2 minute time period after phototobleaching is depicted. Some fast retrograde vesicles are indicated with arrows. Scale bar is 10 μm.

To further demonstrate that expression of Lis1 RNAi indeed disrupts retrograde transport of Nrg vesicles in fully developed GF, we co-expressed UAS-Lis1^RNAiH^ and UAS-Nrg^eGFP^ in mature GFs with GF-Split-Gal4 and live imaged the GF axon. GF-Split-Gal4 is a weaker driver than the R68A06 line and with the expression of only one copy of UAS-Nrg^eGFP^ most fast anterograde and retrograde vesicles are below resolution. However, imaging at one frame per second allows to track the slow retrograde moving vesicles in a large portion of the axon (65 μm) over a long time period (10 min) very well (Fig 5a). In a wild type background most of these slow retrograde Nrg^eGFP^ vesicles moved through the entire bleached area during the imaging period (S3 Movie, Fig 5a). In contrast, when Lis1 expression was knocked down, most Nrg^eGFP^ vesicles did not enter or did not cross the entire bleached area during the 10 min imaging (S4 Movie, Fig 5a, right panel). When we quantified the numbers of Nrg^eGFP^ vesicles entering the bleached area, there was a significant reduction of retrograde vesicles in the Lis1 knock down background, when compared to wild type controls (Fig 5b, right panel) and they moved significantly slower (Fig 5b, middle panel). In addition, we assessed stationary Nrg^eGFP^ vesicles as visualized by the vertical projections in the unbleached region of the kymographs and found that their numbers were increased five-fold when UAS-Lis1^RNAiH^ was co-expressed (Fig 5b, right panel). This demonstrates that Lis1 is required for retrograde transport of slow Nrg^eGFP^ vesicles but that knock down does not fully block retrograde transport.

To also visualize sufficient fast (> 1μm/sec) moving retrograde vesicles we co-expressed Nrg^mCherry^ with UAS-Lis1^RNAiH^ and UAS-Glued^Δ96B^ using the R68A04 Gal-driver. In both mutant backgrounds we observed a significant decrease in flux of fast retrograde (Fig 5c) and anterograde (Fig 5c, Nrg^mCherry^,R68A06 = 6.25 ±0.68, Lis1^RNAiH^ = 1.75 ±0.27, Glued^Δ96B^ = 1.31 ±0.21, p<0.001, Mann-Whitney Rank Sum Test) Nrg^mCherry^ vesicles, in addition to a dramatic increase of stationary vesicles (data not shown). The residual transport of these fast vesicles is likely due to residual Lis1 and Dynactin dimers that do not contain the poisonous Glued^Δ96B^ subunit. However, the reduction of fast moving vesicles suggests that Lis1 and Dynactin are required for transport of fast moving vesicles directly or indirectly as well.

## Discussion

Here, we established the GFs as a novel model to study axon transport that complements live imaging models already established in Drosophila, such as the peripheral nervous system (PNS) neurons in larvae, CNS neurons in developing pupal brains and PNS neurons in the adult wing because it provides a distinct perspective [62–64]. Due to its ~10x larger axon it has excellent signal to noise ratio during imaging allowing to visualize slow and small fast moving vesicles in an individual axon. Both GFs run in parallel, which can be imaged simultaneously and injured (or dye-injected) individually with electrodes. Thus the GFs are a unique new model that will allow to study CNS-specific axon transport or injury signaling as well as slow degenerative diseases and aging phenotypes as the axons can be imaged in young and old flies.

When we analyzed axon trafficking of Nrg^eGFP^ and Nrg^mCherry^, we found with both constructs that anterograde vesicles moved faster than retrograde vesicles and that there are at least two major populations of retrograde vesicles, slow vesicles that moved with varying speed and fast vesicles that moved mostly with consistent speed. In Drosophila, different types of cargoes and vesicles with distinct purposes have been described to move with different velocity. Retrograde Wit (1.55 μm/s), Tkv (1.36 μm/s), BDNF (1.1 μm/s), Rab 3 (1.09 μm/s) and Rab19 (1.07 μm/s) vesicles move with higher velocity than retrograde APP and Rab7 vesicles that have an approximately mean velocity of 0.5 μm/s [22, 65–68]. Thus, it is conceivable that slower (~ 0.4 μm/s) and faster (~ 1.3 μm/s) Nrg vesicles are transported in different type of vesicles and therefore may serve distinct functions.

However, when we compared Nrg^eGFP^ and Nrg^mCherry^, we also observed two main differences between the constructs. While a similar amount of vesicles moved in both directions with Nrg^mCherry^, significantly more retrograde than anterograde vesicles were visible with Nrg^eGFP^. This seems not to be Nrg-specific, as we have observed the same phenomenon with GFP-tagged constructs of other numerous proteins such as wishful thinking, breathless and lysosomal-associated membrane protein 1 (unpublished data). A second difference was that we found more slower and less faster retrograde vesicles with Nrg^eGFP^ than Nrg^mCherry^. The differences may be due to that either the biochemical properties of mcherry and GFP tags may allow for preferentially better visualization of different types of vesicles or the tags affect Nrg trafficking distinctively, or a combination of both. In addition to numerous other aspects, mCherry and eGFP differ with respect to photo stability, pH-sensitivity, hydrophobicity, chromophore maturation and their ability to forms dimer [69–71].

Although anterograde Nrg^eGFP^ are present, most of them were fainter than Nrg^mCherry^ and barely visible in the Kymographs. Therefore, the visible anterograde flux of Nrg^eGFP^ is likely to be lower than the actual flux, which increases the relative proportion of retrograde vesicles of all vesicles imaged. Comparison of multiples studies suggests that in vitro maturation is faster than in vivo maturation, that different GFP variants (e.g. GFP, sfGFP, GFPmut3, eGFP,) mature at different rates and maturation rate is different in different bacteria strains and eukaryotic cells [71, 72]. While in a cell-free system eGFP has been reported to mature faster than mCherry [73], the half-time maturation rate in E. coli at 37°C is somewhat similar for mCherry and eGFP with 15 min and 14 min, respectively [74, 75]. However, two mutations in eGFP were introduced to optimize the folding of the much slower maturing native GFP at 37°C. Therefore, a possible explanation is that in flies raised at 25°C eGFP is less effective than mCherry. An alternative or an additional factor for lower visibility of eGFP in anterograde vesicles may also be that GFP is much more pH sensitive than mCherry [69] but both tags were attached to the cytosolic C-terminus of Nrg [11, 49]. With respect to differences in retrograde transport, eGFP and eGFP fusion proteins have been shown to inhibit polyubiquitination [76] and in contrast to mCherry, eGFP forms weak dimers [69]. These aspects may be contributing factors that affect Nrg sorting to slow or fast moving retrograde vesicles. Even though the tagged constructs may not accurately reflect axonal trafficking of untagged Nrg protein, we demonstrate that endogenous Nrg accumulates in synaptic terminals when retrograde transport is inhibited using multiple approaches demonstrating that Nrg is a retrograde cargo of synapses.

In Lis1 knock down animals GF synapse formation was disrupted similar to as it has been described for inhibition of Dynactin [44]. In addition, we show that Nrg accumulated in the stunted terminals when the function of either dynein regulator was inhibited. We previously demonstrated that Nrg is critical for GF terminal development and stability of synapses [11–13, 55, 77]. However, retrograde signaling of transforming growth factor beta (TGF-beta), BMP and wingless (WNT) are known to be involved in synaptic differentiation in various model neurons [22, 78–80] and although not directly shown their transport is likely to be affected by down regulation of Lis1 as well. Therefore, the observed functional and morphological GF defects are likely to not only be the result of disrupted retrograde transport of Nrg but also of other signaling or recycling molecules.

Lis1 RNAi phenotypes with the UAS-Lis1^RNAiH^, were not only similar albeit stronger than Lis1 hypomorphic mutants but function, morphology and Nrg accumulation could be rescued, demonstrating specificity and validating this RNAi-line for post developmental knock down experiments. We found that Nrg vesicles also accumulated in full-length GFs in Lis1 knock down animals that were 4 days or 4 weeks old, suggesting that endogenous Nrg is transported from the synapse to the soma throughout adulthood. Surprisingly, in old animals all GF terminals were present and the synaptic function was only mildly impaired, although knock down of Lis1 is expected to not only affect Nrg retrograde transport but all Lis1-dependent cargoes. However, live imaging revealed, that despite a severe disruption of retrograde transport of Nrg^eGFP^ vesicles, there are still many vesicles moving in retrograde direction. Dynein-Dynactin was shown to be sufficient for vesicle transport in vitro [81], but Lis1 promotes dynein binding to microtubules and leads to a persistent-force state critical for the transport of high load cargo [82]. In addition, residual Lis1 in the knock down animals is also likely to be the cause that retrograde Nrg^eGFP^ vesicles were still observed. Nevertheless, despite severe “traffic jams”, commonly associated with degenerative diseases [83, 84], the residual axonal transport in Lis1 knock down animals appears to be sufficient for neuronal function and survival for astonishingly long time periods.

Here, we also show that the Gal4-UAS system in combination with CRISPR can be used to effectively mutate genes cell autonomously in post mitotic neurons. In contrast, to RNAi, CRISPR mutates the DNA but does not affect existing transcripts and the time point of CRISPR induced mutations may vary in individual cells [85, 86]. Thus, CRISPR phenotypes are likely to emerge on a delayed time scale but would be expected, depending on the turnover rate of Lis1 protein, to maximize over time once the gene is mutated. Similar to RNAi knock down animals, Nrg accumulation was seen in all 5 day old GF terminals, suggesting that 5 days are sufficient to induce Lis1 mutations with UAS-Cas9 and UAS-Lis1^gRNA^ using the R68A06 as well as the GF-Split-Gal4 lines but the phenotypic strength may have not reached its full potential at this time point.

In vertebrates and invertebrates, the vast majority of studies involving L1-type CAM functions describe their developmental role at the plasma membrane but they are also associated with learning and memory in the adult [87] and have been recently reported to be imported to the nucleus as well [16–19]. Here, we show that Nrg is retrogradely transported from mature synaptic terminals in the adult. The epitope of the monoclonal antibody BP104 involves the tyrosine of the Nrg FIGQY motif in the ankyrin binding domain and precipitates the unphosphorylated neuronal 180 kDa Nrg isoform (Nrg^180^) [11]. This suggest that full-length Nrg^180^ with an unphosphorylated FIGQY motif is present on the vesicles that accumulate in *lis1* mutants and were destined for retrograde transport from the synapse to the soma. Thus, there are three possible scenarios how retrogradely transported full-length L1-type CAMs may contribute to neuronal function in the adult.

First, Lis1 has been shown to regulate retrograde transport of late endosomes and lysosomes [36, 88]. In this scenario, full-length Nrg is endocytosed at the terminal and transported in a retrograde direction in late endosomal compartments to the soma for degradation or recycling. Therefore, at mature synaptic terminals L1-type CAM levels may need to be carefully regulated and a possible excess derived from L1-type CAMs moving laterally from the axonal membrane to the synapse is down regulated to maintain synapse function or size or may be a mechanism to remove L1-type CAMs from the axon for recycling. Alternatively, Nrg may be part of a “signaling endosome” [22, 89]. Neurotrophins bound to their receptors as well as P2X3 receptors are endocytosed in Rab5-vesicles and are then sorted to Rab7-labeled endosomal compartments that are designated for retrograde axonal transport in vertebrates [20, 23]. Similarly, activated BMP receptors and their ligands are seen to be transported from the Drosophila larval neuromuscular junction to mediate signaling in the soma [22]. L1CAM clustering in the plasma membrane results in ubiquitination and dephosphorylation leading to endocytosis of full-length L1CAM into Rab5-vesicles [90]. L1-type CAMs clustering can also activate ERK 1/2 as well as NF-κB signaling via their interactions with receptor tyrosine kinases and integrins in cis or in trans and the activation of the MAP kinase pathway requires L1CAM internalization [14, 91–93]. Thus it is possible that L1-type CAMs in complex with signaling molecules are transported in a retrograde manner to relay a signal from the synapse to the soma. Finally, Nrg may be a retrograde signal itself. Four recent studies reveal that three different fragments of L1CAM translocate to the nucleus to regulate the expression of c-Myc, NBS1, CRABPII and β-integrins [16–19], which play a role in cell cycle control, differentiation and DNA damage checkpoint response activation linked to chemo- and radio-resistance of tumors [94, 95]. Thus, it is conceivable that some of the retrogradely transported L1-type CAMs are proteolytically processed in the soma and subsequently translocate to the nucleus.

## Supporting information

S1 Movie Nrg^mCherry^ vesicles in GF axons of wild type flies. The GF axon of a UAS-Nrg^mCherry^;R68A06 fly was live imaged in the CvC. After 25 sec photobleaching of a small region of the GF, images were acquired at 4 frames per second. The first minute after photobleaching is shown. Soma is on the left side and the scale is 1 μm.
(MOV)

S2 Movie Nrg^eGFP^ vesicles in GF axons of wild type flies. The GF axon of a UAS-Nrg^eGFP^;R68A06 fly was live imaged in the CvC. After 25 sec photobleaching of a small region of the GF, images were acquired at 4 frames per second. The first minute after photobleaching is shown. Soma is on the left side and the scale is 1 μm.
(MOV)

S3 Movie Nrg^eGFP^ vesicles in GF axons of wild type flies. The GF axon of a GF-Split-Gal4,UAS-Nrg^eGFP^*/+* fly was live imaged in the CvC. After 30 sec photobleaching of a small region of the GF, images were acquired every 2 seconds for 10 min and the corresponding kymograph is displayed in Fig 5a, left panel. Soma is on the left side and the scale is 10 μm.
(MOV)

S4 Movie Nrg^eGFP^ vesicles in GF axons of Lis1 knock down flies. The GF axon of a GF-Split-Gal4,UAS-Nrg^eGFP^, UAS-Lis1^RNAiH^/+ fly was live imaged in the CvC. After 30 sec photobleaching of a small region of the GF, images were acquired every 2 seconds for 10 min and the corresponding kymograph is displayed in Fig 5a, right panel. Soma is on the left side and the scale is 10 μm.
(MOV)
